# Transcription-Replication Collisions—A Series of Unfortunate Events

**DOI:** 10.3390/biom11081249

**Published:** 2021-08-21

**Authors:** Commodore St Germain, Hongchang Zhao, Jacqueline H. Barlow

**Affiliations:** 1 School of Mathematics and Science, Solano Community College, 4000 Suisun Valley Road, Fairfield, CA 94534, USA; 2 Department of Microbiology and Molecular Genetics, University of California Davis, One Shields Avenue, Davis, CA 95616, USA; hozhao@ucdavis.edu

**Keywords:** DNA replication, transcription, R-loops, replication stress

## Abstract

Transcription-replication interactions occur when DNA replication encounters genomic regions undergoing transcription. Both replication and transcription are essential for life and use the same DNA template making conflicts unavoidable. R-loops, DNA supercoiling, DNA secondary structure, and chromatin-binding proteins are all potential obstacles for processive replication or transcription and pose an even more potent threat to genome integrity when these processes co-occur. It is critical to maintaining high fidelity and processivity of transcription and replication while navigating through a complex chromatin environment, highlighting the importance of defining cellular pathways regulating transcription-replication interaction formation, evasion, and resolution. Here we discuss how transcription influences replication fork stability, and the safeguards that have evolved to navigate transcription-replication interactions and maintain genome integrity in mammalian cells.

## 1. Introduction

From bacteria to humans, transcription has been identified as a source of genome instability, initially observed as spontaneous recombination referred to as transcription-associated recombination (TAR) [1]. The link between TAR and DNA replication was established by studies in *Saccharomyces cerevisiae* showing S phase was necessary for TAR [2]. Studies in mammalian cells also showed that TAR was dependent on S phase, further supporting the model that transcription stalled replication fork progression and stimulated recombinational repair and transcription is a major source of endogenous replication stress and DNA damage [3]. TAR can arise from several processes including altering the expression of genes that are required for genome maintenance, the opening of heterochromatin, formation of co-transcriptional structures such as R-loops, or TOP2B cleavage [4,5]. Further, RNAP itself is a large, highly processive enzyme that travels with many accessory factors, similar to the replisome. Physical interactions can occur when the two machineries converge toward the same location or codirectionally as the DNA replication fork moves faster than an elongating RNA polymerase (RNAP) [6,7].

Many components of DNA replication are conserved throughout all kingdoms of life, however there are key differences between organisms that influence how active forks interact with transcriptional complexes. All forms of life replicate DNA using bi-directional replication forks, but the placement and organization of replication origins differ widely between bacteria, yeast and mammals. In the bacterium *E. coli*, there is single origin of replication, and this one-bidirectional fork replicates the entire ~4 Mb circular genome. In the budding yeast *S. cerevisiae*, about 400 autonomously replicating sequences (ARS) narrowly define where the replication origins are located [8]. In mammals, the locations of replication origins are less rigidly defined and thought to be stochastic. Helicase activity also differs from prokaryotes and eukaryotes. The replicative helicase in bacteria is tethered to the lagging strand and moves 5′ to 3′ while in eukaryotes and archaea the helicase is bound to the leading strand and moves 3′ to 5′ [9]. These fundamental differences influence the location, formation, and resolution of transcription-replication interactions (TRIs) in single and multicellular organisms.

## 2. TRI Directionality and Consequences on Genome Stability

### 2.1. Codirectional versus Convergent TRIs

Studies in bacteria demonstrated that the direction of transcription relative to replication at TRIs has a profound effect on genome instability. Convergent TRIs have stronger effects than codirectional interactions [2]. In a set of experiments where 25 bacterial species were studied, 67–100% of their essential highly expressed genes are located on the leading strand which results in codirectional movement of transcription and replication [10]. Another study was performed investigating convergent TRIs between RNAP and replication forks in *E. coli* [11]. Here the authors show that when a replication fork is stalled by a convergent RNAP, a transcription-coupled repair protein, Mfd, removes the stalled RNAP and helps restart the replication fork. 

Codirectional TRIs can also cause replication stress. Replication forks encountering an RNAP moving in the same direction are associated with nonsynonymous mutations, though the effects were less pronounced than convergent collisions [12]. One reason why codirectional transcription may still interfere with replication is the formation of R-loops. R-loops occur co-transcriptionally and are enriched in areas of strand bias in the distribution of guanines and cytosines (GC-skew) [13]. G-rich RNA bound to C-rich DNA is more stable than dsDNA, therefore R-loops may pose as an obstacle for replication fork progression behind an elongating RNAP [14]. However, codirectional TRIs only have modest effects on replication fork progression compared to head-on events [15]. Here, in vitro studies in *E. coli* found that a replication fork approaching RNAP moving codirectionally uses an mRNA transcript to continue replication after the RNAP has been displaced. This suggests that the stalled replication fork can restart at the same location without first having to cleave the DNA. 

### 2.2. Distinct Consequences of Convergent and Codirectional Collisions

Convergent TRIs are more mutagenic than codirectional TRIs in B. subtilis, resulting in elevated levels of genomic rearrangements and gene fusions [12]. Genes encoded on the lagging strand were found to contain more point mutations than genes on the leading strand. Higher expression and longer gene length correlated with increased accumulation of mutations. The type of damage to the DNA may also be dependent on the orientation of the TRI. A construct was designed in B. subtilis to create convergent and codirectional TRIs using the same promoter and gene and damage was measured [16]. The authors found that substitutions in the promoter regions are a result of a convergent TRI, while indels are from replication-stalling events triggered by TRIs where the replication fork first encounters the transcriptional machinery. Both codirectional and convergent TRIs experienced damage in the form of indels and base substitutions throughout the gene but were strongly enriched in the promoter region. This, together with the results that promoter indels are closely associated with changes in gene expression may suggest that TRIs could lead to gene expression changes [17].

The observation that the lagging strand experiences an increased mutation rate may arise from the fact that discontinuous synthesis uses multiple RNA primers and different polymerases. The primers must be removed, then the DNA is ligated together at these Okazaki junctions; sequences at these junctions carry a high mutation rate. Nucleosome and transcription factor binding to these locations increase the mutation frequency because they are impediments for DNA polymerase which removes the RNA primer along with the initiator DNA that was first synthesized by the error-prone DNA polymerase [18]. Thus, increased mutation on the lagging strand may arise from a combination of TRIs and error-prone processing of Okazaki fragments.

## 3. Causes of TRI Formation

### 3.1. Topological Constraints of TRIs

**Figure 1:**
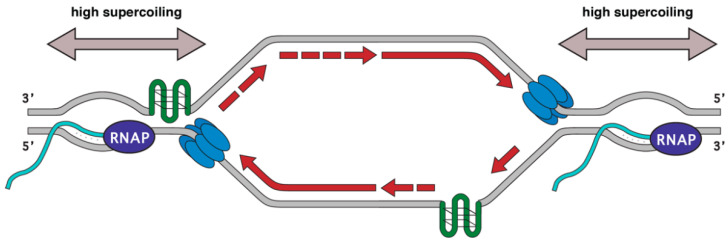
Factors influencing transcription-replication interactions. The orientation of RNAP, secondary structure formation, and DNA topology strongly influence the impact of transcription on replication-associated genome instability. A bidirectional replication origin is in the center with the left replication fork moving toward a convergent RNAP and the right fork moving toward a codirectional RNAP. TRIs are enriched for multiple factors associated with replication stress, including R-loops (turquoise) and DNA sequences forming G-quadruplex (G4, green) structures. R-loops themselves may pose a roadblock for replication but may also tether RNAP to the template strand. Duplex unwinding by the MCM2-7 helicase (blue) or RNAP (violet) can lead to excessive supercoiling ahead of the replication fork requiring topoisomerase activity to relieve torsional strain. Genomic regions with multiple replication destabilizing factors likely increase the chances for fork stalling/collapse, whether they occur on opposite strands (left) or sequentially (right).

During replication and transcription, DNA accumulates positive supercoils in front and negative supercoils behind the translocating machinery. If replication forks encounter an RNAP moving codirectionally, the RNAP would have a trailing negative supercoil while replication forks would be generating positive supercoils and the net change would be zero. However, if replication and transcription are converging toward each other this would cause the intervening section of DNA to experience positive supercoiling from both processes (Figure 1). To alleviate the torsion during replication, the type I and type II topoisomerases TOP1 and TOP2 travel with moving forks relieving topological stress [19]. TOP1 creates breaks in a single strand of DNA while TOP2 creates nicks in both strands of DNA [20]. Topoisomerase activity is also required for proper gene expression and this effect is enhanced in the context of chromatin. In vitro, Topoisomerase II is needed for full RNA synthesis when the DNA is bound to nucleosomes [21]. Eukaryotic TOP1 also helps regulate gene expression by accelerating elongation in vitro [22]. These observations were recapitulated in vivo: TOP1 physically associates with the transcriptional machinery, and its activity is required for efficient elongation and promotes RNAP pausing [23]. Thus, both type I and type II topoisomerases promote efficient transcription in the context of chromatin where additional torsional stresses may be at play. It is possible that extremely supercoiled DNA created by converging replication and transcription may be a poor substrate for the topoisomerases due to its structure or molecular crowding. TOP1 depletion in human cell lines leads to the accumulation of the DNA damage marker γH2AX at transcription termination sites while depletion of the splicing factor SRSF1 does not [24]. These same termination sites are also enriched for DSBs by i-BLESS. Thus, loss of TOP1 recruitment may prolong replication fork stalling induced by excess supercoiling, leaving stalled forks and associated ssDNA vulnerable to nucleolytic attack.

### 3.2. TOP2B and DNA Damage

An increase in transcriptional activity has been shown to elevate DNA damage, with most studies attributing to the cause of damage to conflicts between transcription and replication [25]. Supporting this, a study on human cells showed that TOP2B is required for androgen-induced gene expression and DSB formation at the TMPRSS2 and ERG genes [5]. Further, translocations form between the two loci in a TOP2B-dependent manner. In the cell line used, androgen induces both replication and transcription, therefore DSBs and rearrangements may arise from TRIs. In normal cells, androgen signaling causes terminal differentiation and further suppression of replication but the rearrangements between TMPRSS2 and ERG still occur. This could mean that DNA damage occurs during the terminal differentiation stage, or that transcription and TOP2B alone are responsible for the DNA breaks and rearrangements. Similarly, specific estrogen-induced genes require transient TOP2B-induced DSBs for proper expression [26]. Transcription occurs throughout the cell cycle to produce RNA and proteins needed for the cell to function. It is difficult to distinguish between transcription-induced DNA breaks and transcription and replication conflict-induced DNA breaks in non-synchronized cells. However, these studies were performed in asynchronous cell cultures, therefore it cannot be ruled out that increased transcription led to enhanced conflicts between replication and transcription machineries leading to DSBs. More recently endogenous DSBs were shown to accumulate at genomic loci also enriched TOP2B, paused RNAP phosphorylated at Ser5, and the DSB repair protein XRCC4 [27]. Intriguingly, these events occurred with a higher frequency at the introns of long genes and correlated with translocation sites. DSBs were formed upon release from the pause site, yet the canonical DNA damage response marker γH2AX was not detected. Altogether these data suggest that DSBs formed as a result of normal transcription could promote the formation of chromosomal translocations.

### 3.3. R-Loops and TRIs

R-loops are three-stranded DNA structures created when RNA anneals to a complementary DNA strand forming an RNA:DNA hybrid that displaces the non-complementary DNA strand as ssDNA (Figure 1). Though transient DNA breathing can allow RNA to hybridize to DNA, co-transcriptional formation of R-loops is more likely because the negative supercoiling directly behind RNAP along with the complementary sequence provides better conditions for hybrid formation [28,29]. They have been implicated in multiple cellular processes including class switch recombination, replication origins, transcriptional regulation, transcriptional termination, and epigenetic regulation [1,13,30,31,32]. A growing body of work has revealed a positive role for RNA:DNA hybrids formed at DSB ends in promoting efficient repair [33]. Excessive R-loop formation also promotes genomic instability, comprehensively reviewed in [33,34,35,36]. Many studies show that defects in RNA metabolism—loss of the RNA:DNA endonucleases RNase H1 and RNase H2, mutation or depletion of RNA splicing factors (THO, TREX, and ASF/SF2), or depletion of mRNA export components (TREX-2) increases both RNA:DNA hybrid levels and markers of DNA damage [34,37,38,39,40,41,42,43,44,45,46,47]. Work in human cells also shows that transcriptional regulation by hormone treatment also induces an increase in R-loops which is correlated with increased DNA damage in S phase as measured by γH2AX [48]. Multiple helicases including Aquarius, Senataxin, DDX21, and DDX1 have also been linked to R-loop removal and replication stress, as deletion or depletion of these factors induces markers of DNA damage including DNA repair protein focus formation concomitant with increased RNA:DNA hybrid levels [40,49,50,51,52]. However, mutations interfering with transcription-like pre-mRNA splicing impact cellular function on many levels; defects in splicing impact gene expression, chromatin structure, and cell cycle progression [53,54,55,56]. Alterations in RNA metabolism can also affect removal of RNA primers on Okazaki fragments, potentially causing problems with replication and genome stability [57]. Thus, the pleiotropic nature of these mutations makes disentangling, the role R-loops play in genome instability, challenging. Techniques inducing rapid and specific protein depletion such as Auxin-inducible degradation or Trim-Away, both of which employ ubiquitin-mediated proteasomal degradation, that could narrow the window of time proteins are absent, and help separate direct vs. indirect consequences on R-loops and genome instability [58,59]. 

### 3.4. RNAP Association with TRIs

The vast majority of R-loops are co-transcriptionally formed; therefore, it is challenging to separate the effect of RNAP and RNA:DNA hybrids on TRIs. However recent studies provide exciting clues to how R-loops and RNAP may work independently or together to disrupt replication fork progression. When analyzing transcription-replication conflicts in vitro, R-loops on the leading-strand template pose less of a problem than RNAP alone while R-loops on the lagging-strand template are insignificant replication blocks. When comparing fork stalling, conflicts with a head-on orientation resulted in a higher percentage of stalled forks over co-oriented conflicts [60]. Persistent RNAP association—such as promoter pause sites—may stabilize RNA:DNA hybrids and impede additional transcriptional complexes from elongating [61]. The opposite may also be true; hybrids could help tether RNAP to the template, further stabilizing the large RNAP complex that is already stably encircled around dsDNA. Both scenarios would result in a stabilized RNAP-RNA:DNA hybrid complex that could act as a replication fork block. We recently developed a novel technique termed transcription-replication immunoprecipitation on nascent DNA sequencing (TRIPn-Seq) to map TRIs genome-wide in primary B cells, identifying ~1000 distinct loci [62]. Intriguingly, these TRI regions were characterized by a bimodal distribution of RNAP, bidirectional transcription, and RNA:DNA hybrid formation. These regions also correlated with an increase in replication protein A accumulation, a mark of replication fork stalling [62]. Thus, it appears that bidirectional promoters RNAP and hybrids present potential roadblocks for both leading and lagging-strand synthesis, strongly increasing the chances to impede replication fork progression. 

RNAP pauses at regulatory sequences at the promoter and within gene bodies, regulating the rate of elongation and gene expression. RNAP can also backtrack when encountering a roadblock or in response to mis-incorporation, creating a stable complex that can halt replication forks and induce DSBs [63,64]. In eukaryotes, the transcription factor TFIIS rescues backtracked RNAP2 molecules by cleaving the RNA, leaving a free 3′ end in the active site for elongation to resume [65,66,67]. Trapping of backtracked RNAP2 by expression of a dominant-negative TFIIS leads to increased pausing at gene promoters and terminators and induces DNA damage [68,69]. These results indicate that transiently backtracked RNAP2 can act as natural polar replication fork barriers at promoters and terminators, however persistent complexes induce genomic instability. It will be interesting to determine if suppression of TFIIS activity leads to increased DNA damage and replication stress in mature cancers. Indeed, overexpression of the negative elongation factor NELFE is associated with tumorigenesis [70]. However, NELFE also suppresses transcription during DSB repair [71]. Thus mis-regulation of negative elongation factors may promote tumorigenesis by altering transcriptional activity, inducing transcription-induced replication stress, and interfering with DNA repair. Stalled and stabilized RNAP molecules may have additional biological consequences. If RNAP remains on the DNA after a TRI, it may hold short tracts of under-replicated DNA together and keep sister chromatids paired until mitosis. Such pairing could be helpful or harmful; it may stimulate recombination-mediated fork restart or exacerbate DNA damage formed during mitosis. Further studies will dissect the direct and indirect ways R-loops and RNAP influence genome instability and TRI formation. 

### 3.5. Ribonuclease H in Bacterial DNA Replication

Aside from its role in ferrying genetic information to ribosomes for protein translation, RNA is involved in regulating gene expression, used as a primer for DNA replication, and numerous other cellular processes. Defective RNA processing has been linked to genome instability such as hyper-mutation and hyper-recombination [72]. Bacterial ribonuclease H (RNase H) specifically hydrolyzes RNA when base-paired to single strand DNA (ssDNA). This activity can occur during replication where RNA primers are used for DNA replication, or at any time during the cell cycle when RNA anneals to DNA. The loss of RNase H activity can cause many different processes to malfunction in a cell. RNase H can cleave transcripts which act as primers at the origin of replication for the *E. coli* plasmid ColE1 [73]. Although ColE1 can still be replicated, the replication origin was no longer in the same location when RNase H was not present in the reaction [74]. These results suggest that RNase H has a role in establishing replication origin location and suppressing ectopic replication initiation events. This idea was supported by studies of the pBR322 plasmid, a ColE1-type plasmid which normally requires DNA polymerase I for plasmid replication in *E. coli*. Intriguingly, the pBR322 plasmid can still replicate in the absence of DNA polymerase I when RNase H activity is also suppressed. Under these conditions, pBR322 replication requires transcription at oriC, the origin of replication in the *E. coli* genome [75]. 

The idea that RNase H is involved in establishing precise replication origins is also supported by studies where the oriC is deleted from the *E. coli* genome [76]. The authors hypothesized that oriC deletion would inhibit DNA replication and be lethal. Instead, cells lacking oriC can still replicate in RNase H-defective cells. Their findings show at least four other sites can be used as replication origins in the absence of both oriC and RNase H activity. These sites, termed OriK, were found to form persistent DNA:RNA hybrids (R-loops) only in the absence of RNase H, which enabled replication to initiate from these ectopic locations [31]. As a result of this unusual replication origin usage, multiple replication forks are formed during replication and convergent replication forks merge similar to eukaryotic systems. Under normal conditions, RNase H suppresses these ectopic replication origins by hydrolyzing and removing the R-loops formed at oriK sites. The R-loops may be providing a primer for DNA polymerase to extend from, it could be stabilizing the three stranded nucleic acid structure for replication proteins to bind, or most likely doing both.

### 3.6. Eukaryotic RNase H and Replication Fidelity

In eukaryotes, there are two types of RNaseH: the monomeric RNase H1 and heterotrimeric RNase H2. The three RNase H2 subunits form a stable complex, and all three are required for function [77,78]. Both RNase H1 and RNase H2 can process R loops, however, only RNase H2 also cleaves embedded ribonucleotides (rNMPs) from duplex DNA termed ribonucleotide excision repair (RER) [79]. Mis-incorporation of rNMPs arises during replication initiation by RNA primases and elongation by DNA polymerases, and can also cause replication stress and DSB breaks if not properly processed [80,81,82]. Depletion of RnaseH2 but not RNase H1 results in hypersensitivity to alkaline treatment, confirming RNase H2 activity is required for rNMP removal [80,83,84]. In humans, mutations in all three RnaseH2 subunits are associated with Aicaridi-Goutières syndrome (AGS), an autosomal recessive disorder affecting the skin, immune system, and brain often leading to severe neurological dysfunction [85]. Cells depleted for RNase H2 exhibit a cell cycle progression defect and increased rNMP incorporation, potential causal factors underlying immune system dysfunction in AGS [86]. Unprocessed rNMP incorporation is also thought to be the cause of RNaseH2 embryonic lethality [84,87]. No other known R loop processing factors are associated with AGS disease, therefore rNMP incorporation is the likely culprit even if R loops play a role [88,89]. Indeed, a study modeling multiple AGS-related mutations in yeast exhibited a partial separation of function—all RER functions were lost while variants could partially complement for R loop removal in functional assays [90]. 

RNase H2 has also emerged as a critical regulator of R loops in eukaryotes. It interacts with PCNA via PIP box motif, suggesting an important role during replication [77,91]. Its association with DNA is also cell cycle regulated with enhanced chromatin association in S and G2 [92]. Intriguingly, G2 but not S phase expression of RNase H2 rescued the R loop-induced rapid senescence of cells lacking telomerase and Rad52. Telomeres replicate late, therefore these results suggest that RNaseH2 plays a key role in processing replication-associated R loops sensed in G2. However, this timing also coincides with its RER function, as rNMPs incorporated during replication need to be repaired before the next cell cycle. In contrast, RNaseH1 is not regulated in a cell-cycle-dependent manner but does respond to high R loop loads and interacts with the ssDNA-binding protein RPA [92,93]. This interaction may help recruit RNase H1 to abnormal R loops associated with replication stress and DNA damage events. But how and why RNaseH1 primarily responds to high levels of abnormal R loops still needs further exploration. It is possible its interaction with RPA is regulated by additional factors or post-translational modification, relegating it to a back-up role. 

## 4. Genomic Loci Experiencing Transcription-Associated Replication Stress

### 4.1. Fragile Site Instability and Oncogene Overexpression

Chromosomes contain genomic loci prone to recurrent damage called fragile sites and were originally mapped cytogenetically by fluorescence in situ hybridization (FISH) on metaphase chromosome spreads. Fragile sites can be deleterious because they accumulate deletions and rearrangements spanning large genomic regions. This could lead to tumorigenic mutations; indeed, fragile site damage and structural variations have been observed in many cancers [94]. There are two different types of fragile sites: early replicating fragile sites (ERFSs) and common fragile sites (CFSs) [95]. Both ERFS and CFSs are defined by their replication timing, the accumulation of recurrent tissue-dependent damage, and sensitivity to replication stress. CFSs have been studied since their discovery in the late 1970s but are still poorly understood. Analysis of CFSs shows that the areas contain long genes, are origin poor, are adenine and thymidine rich, and often under replicated [95,96,97]. CFSs were found in cells experiencing replication stress using aphidicolin, a DNA polymerase inhibitor. Early replicating fragile sites were discovered in 2013 by Barlow and Faryabi et al. [95]. There are many more putative ERFSs (619 sites) than there are CFSs, however less extensive testing has not revealed the frequency of breakage at vast majority of potential ERFSs [95,96,98,99]. ERFSs characteristics differ from CFSs in that they are located in early replicating regions as the name suggests, origin-rich and gene rich, enriched for CpG islands, and have a high GC content. 

Although ERFSs have different genetic and epigenetic characteristics from CFSs, they may share similar properties conferring fragility. Transcription has been implicated in DNA breakage at both ERFSs and CFSs, potentially explaining the tissue dependence of fragile site breakage [95,100,101]. CFS damage is hypothesized to arise from under-replicated DNA persisting into mitosis, forming DNA bridges in anaphase [102,103,104,105]. Indeed, TRIs could induce replication fork stalling leading to the under-replication of DNA in these regions. However recent studies indicate that transcription across very large genes affects replication timing and genome instability, suggesting that the inability to complete replication may be more related to changes in replication timing than transcription-replication collisions [106,107]. Replication timing also impacts 3D genome organization; and disruption of chromosome contacts can impact origin firing [108,109]. The potential feedback loops between replication timing and chromatin state likely underlie the genome instability observed during oncogene-induced senescence [110]. Though challenging, further studies dissecting replication timing from chromatin state and transcriptional activity are needed to untangle how these factors influence genome instability at fragile sites. 

### 4.2. TRIs in Ribosomal DNA 

There are many highly transcribed copies of ribosomal DNA because of the high number of ribosomes needed to translate all the proteins for different cellular functions. Multiple RNAPs load onto the same locus and transcribe the same region before transcription has been completed by the previous RNAP. Ribosomal DNA encodes sequence-specific replication fork barriers (RFB) to minimize congestion in these high traffic areas. The RFBs allow codirectional movement of RNAP and replication forks and stop convergent replication forks so that they do not collide with the many RNAPs transcribing that region [111,112]. There are increased rates of recombination at these RFBs suggesting that any paused or stalled fork could be damaging to that location [113]. In *S. cerevisiae*, the protein FOB1 acts as a RFB when bound to a specific DNA sequence at the end of each 35S rDNA repeat. FOB1 binding prevents convergent transcription and replication and induces site-specific recombination by promoting DSB formation [114,115,116]. These breaks stimulate the formation of extrachromosomal rDNA circles (ERCs) which are associated with aging. Intriguingly, fob1 mutants have less ERCs and longer life spans [117]. The presence of these RFBs suggest that the convergent replication forks and RNAP would be more deleterious if located within the gene.

### 4.3. Engineered Replication Fork Blocks in Eukaryotes 

A major challenge in studying TRIs is knowing precisely where a replication fork collision occurs. To bypass this problem, researchers have turned to natural systems to engineer site specific replication fork blockages. The Tus/Ter system evolved in *E. coli* as a means to terminate replication forks on a circular molecule [118]. The Ter sequence is a 21 bp sequence and Tus is a protein that binds to that sequence and causes replication termination opposite of the replication origin. The Tus/Ter system has been imported to yeast to investigate the consequences of replication fork blocks. In *S. cerevisiae*, insertion of Ter sequences caused replication fork pausing when a galactose-inducible Tus protein was expressed [119]. This did not cause complete fork arrest but does trigger site-specific recombination. The Ter site is a short sequence, and 3–7 repeats are sufficient to induce fork stalling; this will likely prove a powerful system to investigate the outcomes of replication-transcription interactions in a variety of genomic locations. Since replication origins in yeast are well-defined, it can allow the investigation of fork pausing with high resolution with respect to origins.

The Tus/Ter system has also been used to investigate replication fork stalling in mammals. Using a plasmid that undergoes unidirectional replication and inducible Tus expression, the Tus/Ter system also arrests replication forks in human 293T and mouse embryonic stem cells [120]. Homologous recombination at stalled replication forks may result in long-tract gene conversions and whereas homologous recombination at DSBs does not. Further, BRCA1 regulates homologous recombination at Tus/Ter stalled replication forks, suppressing long-tract gene conversion events at these sites. The precise insertion of Ter sites and flexibility of an inducible promoter for the Tus protein would make the Tus/Ter system very useful in investigating how stalled replication forks are resolved in a variety of chromatin contexts.

### 4.4. Nucleotide Structures Associated with TRIs

Replication forks also stall at DNA sequences able to form into stable non-B structures such as hairpins, cruciform, and G-quadruplexes indicating that certain genomic sequences are intrinsically prone to DNA breakage [121]. Indeed, mammalian fragile sites are enriched for repetitive AT-rich sequences and trinucleotide repeats prone to form secondary structures [122,123]. Poly(dA-dT) tracts can form hairpins that act as replication fork barriers and exhibit genome instability under replication stress in B cells [124]. The size of such repeats can also impact fragility, as placing AT-rich sequences forming cruciform structures promotes DNA breakage in a length-dependent manner [125]. DNA secondary structures such as G-quadruplexes have a higher propensity to form in the presence of transcriptional activity, when elongating RNAP unwinds the DNA duplex creating a more labile and negatively supercoiled ssDNA substrate [126]. G-quadruplex motifs are helical shapes formed in DNA and RNA where guanine tetrads can stack on each other and have been implicated in telomere function, transcription, translation, and genome instability [127,128]. R-loop formation also promotes non-B structure formation on the ssDNA non-template strand [129]. Like G-quadruplexes, R-loops are enriched in G-rich areas of the genome, and recent findings show that these two structures act synergistically to increase transcription [129]. Both R-loops and G-quadruplexes correlate with lower nucleosome occupancy, CpG islands, and open chromatin all of which are associated with replication problems [130,131]. Indeed, over 90% of native TRIs in B lymphocytes overlap sequences capable of forming G-quadruplexes [62]. R-loop formation may also relax negative supercoils, allowing G4 structures to form on the non-template strand [28,132]. However, in B. subtilis, type II topoisomerases promote R-loop formation at HO conflicts, presumably through supercoil resolution [133]. Thus, TRIs themselves may be intrinsically difficult to replicate, which is then exacerbated by replication stress such as low levels of nucleotide pools (Figure 1) [134]. While studies have focused on RNA:DNA hybrids as a source of transcription-replication conflicts, they are just one facet of complex genomic structures that make up TRIs.

## 5. Conflict Avoidance at Transcription-Replication Interaction Sites

### 5.1. Spatio-Temporal Separation of Transcription and Replication

In mammals, origin firing is stochastic and replication timing changes throughout development [135]. Multiple studies mapping replication timing genome-wide show a positive correlation between early replication, gene expression, and chromatin accessibility [135,136,137,138]. This raises a conundrum: why initiate replication in areas of active transcription where TRIs are more likely to occur? New techniques devised to map origin location at high resolution—Okazaki fragment sequencing (OK-Seq), small nascent strand sequencing (SNS-Seq), and high resolution Repli-Seq—reveal transcription influences origin location. Origins are enriched near the start site of active genes, ensuring the co-directional movement of the two complexes [139,140,141]. Transcription appears to play an active role in this process by redistributing Mcm2-7 complexes—the major DNA replicative helicase—away from active gene bodies during G1 [142,143]. This relocation helps maintain genome stability, presumably by suppressing origin firing within active genes and reducing transcription-replication conflicts [143]. Further, MCM complexes are enriched upstream of TSSs, and correlate with firing origins [144]. This arrangement promotes codirectional replication and transcription and reducing more deleterious head-on conflicts. Temporal separation of transcription and replication has also been observed at distinct loci, suggesting mechanisms may regulate the timing of replication and transcription at the single-gene level. When compared to existing replication timing data sets, they found a global anti-correlation between gene expression and replication timing [145]. Examination of individual genes by RT-qPCR showed that histone cluster 1 was replicated late but transcribed early while nearby genes showed the opposite pattern. These findings could also be explained by the replication fork impeding or displacing transcriptional machinery and therefore causing the transcriptional output levels to decrease. However, histone gene transcription is regulated in a cell-cycle-dependent manner, coordinating histone production with genome duplication [146]. Such S phase-regulated loci may experience additional replication timing regulation to limit TRI formation. 

### 5.2. Replication Checkpoint Proteins Mediate RNAP Removal from DNA at Transcription-Replication Conflicts

Since transcription and replication can occur on the same tract of DNA, methods to avoid conflicts between the two machineries have evolved. In eukaryotes, the serine-threonine kinase ATR is an essential DNA damage checkpoint protein in eukaryotes which senses stalled and damaged replication forks, and activates the intra-S checkpoint [147]. ATR also plays a critical role in suppressing TRIs when replication initiation is perturbed. Cells exposed to doxorubicin fire clusters of dormant replication origins in gene-rich transcribing areas, and transcription was suppressed in these neoreplication origin clusters in an ATR-dependent manner [148]. Transcriptional suppression required the degradation of the histone chaperone ASF1, indicating ATR mediates transcriptional suppression by dechromatinizing DNA. 

Similarly, a recent study on yeast found that during hydroxyurea (HU)-induced replication stress, the yeast homolog of ATR Mec1 alleviates transcription and replication conflicts [149]. HU inhibits ribonucleotide reductase and depletes dNTP pools causing the replication machinery to stall [150]. At genomic loci experiencing codirectional transcription and replication, the authors observed a decrease in the amount of chromatin-bound RNAP, however RNAP was retained at sites of convergent transcription and replication. They propose that during HU-induced replication stress, Mec1 phosphorylates the chromatin remodeling complex INO80C and/or the transcription elongation factor PAF1C, leading to the degradation of RNAP at the stalled forks and nearby RNAP by the proteasome at codirectional collisions. This was tested at four independent loci, two with codirectional transcription and replication and two with convergent events. Although the evidence is compelling, much of the decrease in the chromatin bound RNAP could also be explained by RNAP elongation away from the HU-arrested replication fork. since in a codirectional collision, the replication fork would be behind the RNAP. However, genetic evidence places Mec1 in the same pathway as INO80C and PAF1C, suggesting that it plays a more active role in regulating transcription during times of stress. These results are reminiscent of observations in *E. coli* mentioned previously where RNAP and DNA polymerase both dissociate from the DNA at codirectional TRIs [15]. 

Supporting the notion that the intra-S phase checkpoint suppresses genome instability arising from TRIs, phosphorylation of Mrc1 also prevents transcription-associated recombination (TAR) in yeast [151]. Mrc1 can activate Rad53 (the *S. cerevisiae* CHK1 homolog) which then prevents entry into mitosis, upregulates dNTP pools, and activates DNA damage repair [152]. Rad53 activity also functions to release transcribed genes from the nuclear pore complex which may relieve topological stress that likely contribute to genome instability and possibly stalling replication forks at TRIs [153]. Histone 3 methylated at lysine 4 (H3K4me), a mark of active transcription, can slow replication potentially minimizing interactions between transcription and replication. Mutants in RAD53 are hypersensitive to HU, hypothesized to result when cells with stalled replication forks enter into mitosis. When H3K4me was decreased, there was a large increase in the viability of rad53 cells presumably because there were less stalled replication forks [154].

## 6. Resolution of Transcription-Replication Conflicts in Mammals 

### 6.1. DNA Damage Signaling at TRIs 

In mammals, the ataxia-telangiectasia mutated (ATM) kinase is recruited to double-stranded breaks (DSBs) while ATR is recruited to single-stranded DNA that could result from events such as stalled replication forks or resected DSBs [155]. These checkpoint kinases ensure faithful replication and inheritance of the genome. ATM and ATR phosphorylate many targets including CHK2 and CHK1, respectively and these in turn phosphorylate CDC25A to block its interaction with CDK1 which induces a cell cycle arrest to promote DNA damage repair prior to cell division [156,157]. Unrepaired DSBs can lead to unequal segregation of genomic DNA to one or both daughter cells leading to loss of heterozygosity, mitotic failure, cell death, or other problems [158,159]. 

ATR interacts with Mcm2-7, the major DNA replicative helicase, to enforce a checkpoint to minimize the amount of DNA damage carried over from S phase to G2/M—including damage from TRIs [160]. The codirectional or convergent occurrence of TRIs seem to trigger different signaling pathways. A codirectional TRI may lead to an increase in ATM/CHK2 signaling, while a convergent TRI may lead to an increase in ATR/CHK1 signaling [161]. TRI orientation also leads to different R-loop levels as there were less R-loops in a convergent TRI than there were in a codirectional TRI. Another study also shows that ATR is involved in the S to G2 transition [162]. In this study they show that the cell exits S phase as a result of CDK1 phosphorylating FOXM1 and that inhibiting ATR activates FOXM1 prematurely showing another role for ATR helping maintain genome stability. Additionally, the DNA damage checkpoint kinases ATM and DNAPK transiently repress transcription near the sites of DNA damage by evicting RNAP from chromatin in a proteasome-dependent manner, promoting DSB repair [163,164,165]. RNAP removal may be particularly important in homology-directed repair of single-end DSBs generated by collapsed replication forks. Removing RNAP and accessory proteins from chromatin may promote faithful homology search and DNA pairing, suppressing the occurrence of insertion/deletion events.

### 6.2. Nucleases Involved in the Resolution of TRIs

Resumption of DNA synthesis interrupted by deleterious transcription-replication fork interactions often requires nucleolytic cleavage of the stalled fork. In response to replication stress from HU or aphidicolin, replication restart is initiated by cleavage of stalled forks by the structure-specific endonuclease MUS81-EME2 [166,167]. Yet DSBs only became apparent after 18 h, indicating MUS81 cleaves stalled forks after prolonged fork stalling [166]. In contrast, when cells were treated with camptothecin—a topoisomerase inhibitor known to inhibit transcription—DSBs formed were visible within 1 h [168]. Thus, replication stress involving transcription inhibition may result in the rapid appearance of DSBs in S phase. Recently it was reported that replication restart at forks stalled by co-transcriptional R-loops requires MUS81-EME1 [169]. This is intriguing, as EME1 is thought to act predominantly in mitosis [170,171,172].

Artemis and XPF-ERCC1 independently contribute to break formation in response to replication fork stalling, preserving genome stability, and minimizing segregation defects [173]. It was observed that XPF-ERCC1 was not necessary for DSB formation but was required for FANCD2 focus formation [174]. Thus, the nuclease activity promoting DSB formation could be provided by Artemis while ERCC1 recruits FANCD2 to damage sites. XPF-ERCC1 is also necessary to repair DSBs that are adjacent to secondary structures including G-quadruplexes and AT-rich repeats and can remove non-homologous sequences prone to forming secondary structures during recombination-mediated repair [175]. TRIs have a high propensity for G-quadruplex formation, therefore XPF-ERCC1 likely contributes to their resolution [25]. ERCC1-mutant mice also show many age-related phenotypes including shortened lifespans, neurodegeneration, anemia, and stem cell exhaustion linking TRI damage with aging phenotypes [176,177]. 

### 6.3. RECQ5 and BLM Are Helicases Involved in the Resolution of TRIs

RECQ5 is a helicase that has recently been implicated in the resolution of TRIs [178]. RECQ5 is involved in regulating RNAP fidelity at transcribed genes and in preventing stalled replication forks. Lack of RECQ5 leads to the accumulation of RAD18 foci and BRCA1-dependent RAD51 foci. RAD18 is responsible for the ubiquitination of stalled replication forks [179]. RAD51 is the central protein in homologous recombination and is involved in homology search and DNA strand exchange of broken ends of DNA [180]. This could mean that lack of RECQ5 leads to DNA damage or that when it is present, it has a function to minimize the accumulation of these DNA damage repair proteins. In another study it was shown that CDK1 phosphorylates RECQ5, promoting MUS81-EME1-mediated cleavage of replication intermediates at CFSs in G2/M [181]. It was suggested that RECQ5 alleviates the accumulation of RAD51 which then allows access for MUS81-EME2 to cleave stalled replication forks. In a separate study the authors showed that RECQ5 bound to RNAP2 is responsible for conjugating SUMO2 to PCNA, another non-helicase role for RECQ5 in TRI resolution [182]. This leads to the enrichment of CAF1 and histone H3.1 which destabilizes RNAP2 bound to chromatin and allows replication fork progression. 

Cell cycle arrest induced by ATR promotes the resolution of transient transcription-replication conflicts [183]. Here, loss of DNA repair proteins FANCD2, BLM, and BRCA2 led to DNA damage in mitosis, chromosome instability, and cell death indicating that homologous recombination mediates repair. The BLM helicase may also help unwind complex secondary structures formed during transcription, as it suppresses genome instability in areas that contained G-quadruplex motifs in transcribed genes [184]. Together, these observations set up a model where following the occurrence of a TRI, ATR kinase is activated, recruiting a nuclease such as XPF-ERCC1, MUS81-EME1/2, or Artemis to cleave the stalled replication fork. Artemis has been known to cleave AT-rich sequences while XPF-ERCC1 cleaves AT-rich and G-rich secondary structures [175,185]. Next a helicase such as RECQ5 or BLM would unwind secondary structures such as hairpins, G-quadruplex, or R-loops. The resulting DSB can then be repaired by HR or NHEJ, depending on the structure of the broken DNA ends [186]. 

### 6.4. Concluding Remarks

There is still much to understand concerning TRIs in mammalian cells. Work in cell lines and with ectopic DNA constructs have revealed mammalian-specific factors in TRI sensing and resolution, however the vast majority of these experiments have been performed in immortalized cell lines—the most commonly used are HEK293 and HeLa cell lines—which are aneuploid, replicate indefinitely, and have different cell cycle control gene expression profiles thus they may not be informative when investigating unperturbed chromosome biology [187,188]. Many of these factors likely directly affect where and how transcription-replication conflicts arise, as well as the mechanisms involved in their resolution. Thus, many important questions concerning TRI formation remain to be answered in primary cells: What are the consequences of TRIs on genome instability, and where do TRIs naturally occur (Figure 1)? Defining the locations of endogenous transcription-replication interactions will be critical to dissecting how TRIs influence genome instability and the roles they play in human disease and aging.
